# Association between MTHFR polymorphisms and vitamin D status in infertile women: a mediation analysis

**DOI:** 10.3389/fnut.2025.1644302

**Published:** 2025-09-11

**Authors:** Ruiqiong Zhou, Zhenghong Zhu, Zhaoyi Wang, Mei Dong, Li Huang, Songlu Wang, Xiqian Zhang, Fenghua Liu

**Affiliations:** ^1^Center for Reproductive Medicine, Guangdong Women and Children Hospital, Guangzhou, China; ^2^Women and Children’s Hospital, Southern University of Science and Technology, Shenzhen, China; ^3^School of Public Health, Sun Yat-sen University, Guangzhou, China

**Keywords:** MTHFR polymorphism, vitamin D, homocysteine, infertility, mediation analysis

## Abstract

**Background:**

Methylenetetrahydrofolate reductase (MTHFR) regulates folate metabolism and homocysteine (Hcy) methylation. Impaired folate metabolism and vitamin D deficiency are both closely associated with female reproductive disorders, but their specific roles and relationship remain largely unknown. This study aimed to investigate the relationship between MTHFR polymorphisms and vitamin D status and to examine the mediating effect of Hcy.

**Methods:**

A total of 6,344 infertile patients were included in this retrospective study. Multivariable logistic regression and multiple linear regression models, and stratified analyses were used to investigate the relationship between MTHFR polymorphisms (C677T and A1298C) and vitamin D status. Smooth curve fitting model and spearman correlation analysis were used to explore the correlation between Hcy levels and vitamin D status. Mediation analyses were performed to examine the direct and indirect effects of MTHFR polymorphisms on vitamin D status.

**Results:**

The risk of vitamin D deficiency and serum Hcy levels were significantly higher in patients with MTHFR677CT and TT compared with CC (*p* < 0.001 for both). In multivariate regression models, MTHFR677CT and TT were positively associated with vitamin D deficiency compared with CC. No significant differences were found for A1298C polymorphism. Smooth curve fitting models showed that serum Hcy was linearly correlated with both 25(OH)D levels (*p*-nonlinear = 0.063) and prevalence of vitamin D deficiency (*p*-nonlinear = 0.261). In mediation analyses using logistic regression models, Hcy mediated 15.8 and 41.6% of the associations between 677CT and TT (versus CC) and vitamin D deficiency, respectively.

**Conclusion:**

The effect of C677T polymorphism on vitamin D status can be explained jointly by a direct association between C677T polymorphism and vitamin D, and an indirect association mediated by Hcy.

## Introduction

Methylenetetrahydrofolate reductase (MTHFR) in a key enzyme in the folate pathway, responsible for folate metabolism and homocysteine methylation (1). C677T and A1298C are the two most common polymorphisms of MTHFR gene (2, 3). Mutations at these two loci reduce MTHFR enzyme activity, resulting in disruption of the conversion of 5,10-methylenetetrahydrofolate to 5-methylenetetrahydrofolate and cysteine to methionine, thereby causing disturbances in folate and homocysteine status (4). The MTHFR C677T mutation results in the conversion of alanine to valine at position 677, leading to a decrease in MTHFR enzyme activity, with only about 30% of the enzyme activity retained in homozygous TT genotype (3). Similarly, the A1298C polymorphism changes glutamate to alanine at position 1,298, resulting in a lesser reduction in enzyme activity (5).

Folate deficiency or MTHFR gene defects exhibit DNA hypomethylation and abnormal biochemical and/or phenotypic changes in animal models (6, 7), cell culture (8) and humans (9–12). Currently, MTHFR gene testing is widely used for clinical diagnosis of folate metabolism capacity and to provide a reference for the use of medications such as folic acid before or during pregnancy. Many studies have reported an association between MTHFR C677T and A1298C polymorphisms and recurrent pregnancy loss (RPL) (13–16). In addition, MTHFR C677T and A1298C polymorphisms have been associated with a variety of pregnancy-related complications (17–19), such as preeclampsia. The relationship between MTHFR polymorphisms and IVF/ICSI outcomes has also been explored, but with conflicting results (20–22). MTHFR polymorphisms are associated with female reproductive health, but their specific roles and mechanisms remain largely unknown.

Mutations in MTHFR gene result in elevated homocysteine (Hcy) levels (4). Hcy is an intermediate metabolite of methionine, and its elevation is associated with pro-oxidative and pro-thrombotic states that induce endothelial dysfunction, thus damaging various organs and tissues (23, 24). Although MTHFR polymorphisms and/or hyperhomocysteinemia are strongly associated with cardiovascular risk and adverse pregnancy outcomes, studies have shown that lowering Hcy levels by increasing folic acid intake is not necessarily effective in reducing the risk of adverse outcomes, or may even yield opposite results, suggesting that other factors may interfere with folate metabolism pathway and influence its downstream effects (25–28).

Vitamin D deficiency is a health concern for women worldwide and is associated with cardiovascular disease, cancer and all-cause mortality (29, 30), as well as adverse pregnancy outcomes and female reproductive diseases (31–34). However, the role of vitamin D and the mechanism by which it is associated with these disease are unclear. Vitamin D works by activating the vitamin D receptor, which regulates the transcription of target genes responsible for various biological processes (35–37). In addition to its well-known role in calcium balance and bone health, vitamin D regulates cell proliferation and differentiation, apoptosis, angiogenesis, anti-inflammation, immunomodulation, and multiple metabolic pathways (38–40).

The relationship between folate metabolism and vitamin D remains poorly understood. Previous studies have suggested that folate may affect bone health, which may be related to vitamin D function (41), and that there may be an association between MTHFR polymorphisms and bone mineral density (42). In vitamin D-deficient mice, supplementation with folic acid, vitamin B12 and vitamin D together may improve learning and memory performance more than vitamin D alone (43). Studies have shown an inverse relationship between 25-hydroxyvitamin D [25(OH)D] and Hcy levels in the general population (44), and that both vitamin D deficiency and hyperhomocysteinemia are risk factors for cardiovascular disease (23, 30). One study has investigated the effect of MTHFR C677T polymorphism on serum vitamin D and Hcy levels in women with RPL, but this study had a small sample size (*n* = 837) and included only women with RPL, which may not be generalizable to other populations (45).

We hypothesized that MTHFR polymorphisms may be associated with vitamin D status; however, no studies have examined the specific nature of this relationship or quantified the strength of the association. Therefore, we genotyped the MTHFR C677T and A1298C polymorphisms in infertile women and investigated their relationship with vitamin D status, aiming to better understand how and to what extent the MTHFR polymorphisms affect vitamin D status and hopefully providing a theoretical basis for individualized treatment of infertility.

## Methods

### Study design and participants

Infertile patients undergo a comprehensive infertility evaluation at our fertility center, including infections, endocrinology, metabolism, ultrasound, and other tests related to pregnancy preparation/infertility including MTHFR gene polymorphisms, homocysteine and vitamin D, as well as semen analysis. All patients are informed of the benefits and costs of each test, and it is up to the patient to decide whether or not to undergo infertility-related tests. The study was approved by the Institutional Review Board of Guangdong Women and Children Hospital. Given the retrospective design, the requirement for informed consent was waived in accordance with institutional and national ethical guidelines. The study included infertile patients who underwent a comprehensive infertility assessment between January 2019 and May 2024, which included testing for MTHFR gene polymorphisms, serum Hcy and 25(OH)D levels. In this study, we included infertile patients who had started taking folic acid supplements in preparation for pregnancy. We excluded patients from the study if they met any of the following criteria: use of hormone therapy; vitamin D and calcium therapy within 3 months; uterine abnormalities; other medical conditions including kidney disease, hypertension, diabetes and tumors; and missing core data. According to international guideline recommendations (46–48), patients were divided into two groups according to the criteria of serum vitamin D deficiency: < 50 nmoL/L group and ≥ 50 nmoL/L group.

### Measurement of biochemical parameters

Blood samples were collected from patients at their first visit to the fertility clinic to assess biochemical parameters. All tests were performed by our clinical laboratory in a timely manner. Serum 25(OH)D, anti-mullerian hormone (AMH), and fasting insulin were assessed using chemiluminescence. Serum Hcy, fasting glucose, triglyceride and total cholesterol were measured by enzymatic method. Serum low-density lipoprotein, high-density lipoprotein, and hemoglobin were detected using colorimetric method.

### MTHFR gene polymorphisms

DNA extraction kit (Magen, Guangzhou, China) was used to extract genomic DNA according to the manufacturer’s instructions. Genotypes for the MTHFR C677T and A1298C loci were determined by fluorescence quantitative polymerase chain reaction, as previously reported (19). MTHFR C677C, C677T and T677T were determined as wild-type (CC), heterozygous (CT) and homozygous (TT), respectively; MTHFR A1298A, A1298C and C1298C were determined as wild-type (AA), heterozygous (AC) and homozygous (CC), respectively.

### Statistical analysis

Statistical software package (SPSS, version 22.0) and R software (version 4.3) were used to perform the analyses. Kolmogorov–Smirnov test was used to determine whether the continuous variables were normally distributed. Continuous variables were expressed as mean with standard deviation or median with interquartile range, and comparisons of differences between two groups were made using Student’s *t*-test or Mann–Whitney *U*-test, and comparisons of differences between three groups were made using one-way ANOVA or Kruskal-Wallis test, as appropriate. Categorical variables were presented as number with percentage and compared by Pearson’s chi-square test or Fisher’s exact test. *p*-value < 0.05 was considered statistically significant.

To investigate the relationship between MTHFR gene polymorphisms and vitamin D levels in infertile women, we used logistic regression models to investigate the effect of MTHFR polymorphisms (C677T and A1298C) on vitamin D deficiency and multiple linear regression models to investigate the effect of MTHFR polymorphisms (C677T and A1298C) on serum 25(OH)D levels. The selection of model covariates was screened according to *a priori* clinical and epidemiological knowledge combined with a directed acyclic graph (DAG) (Supplementary Figure S1). Variables were included if they had a *p* value < 0.05 in comparisons stratified by vitamin D levels or MTHFR genotypes, or if they were deemed clinically relevant to vitamin D status (e.g., body mass index (BMI), type of infertility). Two models were built based on DAG when Hcy was the mediating variable. In model 1, minimal sufficient adjusted variables included age, BMI, AMH, type of infertility and causes of infertility to estimate the total effect of MTHFR polymorphisms on vitamin D status. In model 2, age, BMI, AMH, type of infertility, causes of infertility, hemoglobin and season of blood collection were adjusted confounders to estimate the total effect of MTHFR polymorphisms on vitamin D status. To assess the relationships between serum 25(OH)D levels or vitamin D deficiency and serum Hcy levels, smooth curve fitting models were constructed. We used generalized linear model and generalized additive model to explore potential association between vitamin D status and Hcy levels and tested for nonlinearity using maximum likelihood method. The correlation coefficients between Hcy and 25(OH)D were calculated by Spearman correlation analysis for the total population and for different MTHFR C677T genotypes. To ensure data integrity, we included only patients with complete data for the key variables (MTHFR genotypes, serum 25(OH)D levels, and Hcy levels). Missing values for other covariates were imputed using the median.

### Mediation analysis

To examine the direct and indirect effects (via Hcy) of MTHFR polymorphisms on vitamin D status, mediation analyses were performed. We used the classic framework of mediation analysis, based on the three-step approach proposed by Baron and Kenny (49), to assess direct and indirect effects. The following three models were constructed:

1. Total effect of MTHFR polymorphisms on vitamin D status:

$$Y=\beta_{0}+\beta_{1}X+\beta_{2}C+\in_{Y}$$

Here, Y represents vitamin D status, X represents the MTHFR polymorphisms, C denotes a set of covariates controlled for in the model, *β_1_* is the coefficient of the total effect of MTHFR polymorphisms on Vitamin D, and *ϵ* is the residual error term of the model.

2. Effect of MTHFR polymorphisms on Hcy:

$$M=\beta_{0}+\beta_{3}X+\beta_{4}C+\in_{M}$$

In this model, M represents Hcy levels, and *β3* is the coefficient estimating the effect of MTHFR polymorphisms on Hcy. C represents a set of covariates controlled for in the model.

3. Direct effect of MTHFR polymorphisms on vitamin D status (controlling for Hcy):

$$Y=\beta_{0}+\beta \prime_{1}X+\beta_{5}M+\beta_{6}C+\in_{Y}$$

Here, *β’*_*1*_ represents the direct effect of MTHFR polymorphisms on Vitamin D status, and *β5* denotes the effect of Hcy on vitamin D status, controlling for covariates in C.

Based on the mediation analysis framework, the indirect effect of MTHFR polymorphisms on Vitamin D status (via Hcy) and the mediation proportion can be calculated using the following formula:


$$\text{Indirect Effect}=\beta_{3}*\beta_{5}$$

*For continuous outcome* var*iables:*


$$\text{Proportion Mediation}=\frac{\text{Indirect Effect}}{\text{Total Effect}}=\frac{\beta_{3}*\beta_{5}}{\beta_{1}}$$

*For binary outcome variables:*


$$\;\text{Proportion Mediation}=\frac{\text{Indirect Effect}}{\text{Total Effect}}=\frac{\left[\exp(\beta_{3}*\beta_{5})-1]*\exp(\beta_{1}^{\prime }\right)}{\exp(\beta_{1})-1}$$

In addition, we calculated the total effect as: $\text{Total Effect}=\beta_{1}$ ; and the direct effect as: $\text{Direct Effect}=\beta \prime_{1.}$

To assess the significance of the indirect effect, we used the bootstrap method, sampling 1,000 times to compute the standard errors and 95% confidence intervals for the indirect effect and mediation proportion. This procedure ensures the robustness and accuracy of the results.

## Results

### Patient characteristics and blood biochemicals stratified according to 25(OH)D levels

A total of 6,344 infertile patients who met the inclusion criteria were included in this study. To explore the features associated with vitamin D deficiency, we divided all patients into two groups according to the threshold of vitamin D deficiency (< 50 nmoL/L). Except for age, Hcy, MTHFR C677T genotype, hemoglobin and season of blood collection, other characteristics of the two groups were comparable (Table 1). The proportion of those aged ≥ 35 years in the 25(OH)D ≥ 50 nmoL/L group was significantly higher than in the < 50 nmoL/L group (31.4% vs. 27.4%; *p* = 0.002). Notably, the MTHFR C677T genotypes were significantly different between the two groups (*p* < 0.001), whereas the MTHFR A1298C genotypes were comparable between the two groups (*p* = 0.176). In addition, Hcy levels were significantly higher in the 25(OH)D < 50 nmoL/L group than in the ≥ 50 nmoL/L group (median 7.5 vs. 7.1 μmoL/L; *p* < 0.001), and hemoglobin levels were significantly lower in the 25(OH)D < 50 nmoL/L group than in the ≥ 50 nmoL/L group (median 132 vs. 133 g/L; *p* = 0.001). There was a significant difference in the season of blood collection between the two groups (*p* < 0.001), suggesting that the prevalence of vitamin D deficiency tends to be higher in winter and spring. BMI, type of infertility, duration of infertility, causes of infertility, AMH, and a range of metabolic indicators were comparable between the two groups (Table 1).

**Table 1:** Patient characteristics and blood biochemicals stratified according to 25(OH)D levels.

Parameters	25(OH)D stratification		
	<50 nmoL/L (*n* = 1,641)	≥50 nmoL/L (*n* = 4,703)	*p-*value
25(OH)D	42.3 (36.0, 46.3)	68.0 (59.2, 80.1)	<0.001
Age ≥ 35 years	450 (27.4)	1,478 (31.4)	0.002
Body mass index	21.4 (19.7, 23.4)	21.5 (19.8, 23.4)	0.225
Type of infertility			0.679
Primary	829 (50.5)	2,348 (49.9)	
Secondary	812 (49.5)	2,355 (50.1)	
Duration of infertility	3.0 (1.0, 4.0)	3.0 (1.0, 4.5)	0.476
Causes of infertility			0.406
Tubal	559 (34.1)	1,625 (34.6)	
Male factor	242 (14.7)	691 (14.7)	
Endometriosis	50 (3.0)	127 (2.7)	
PCOS	84 (5.1)	293 (6.2)	
Other	205 (12.5)	635 (13.5)	
More than one etiology	359 (21.9)	960 (20.4)	
Unexplained	142 (8.7)	372 (7.9)	
Homocysteine (μmol/l)	7.5 (6.5, 8.6)	7.1 (6.1, 8.1)	<0.001
MTHFR C677T			< 0.001
CC (wild type)	816 (49.7)	2,594 (55.2)	
CT (heterozygous type)	661 (40.3)	1720 (36.6)	
TT (homozygous type)	164 (10.0)	389 (8.3)	
MTHFR A1298C			0.176
AA (wild type)	1,000 (60.9)	2,755 (58.6)	
AC (heterozygous type)	557 (33.9)	1,667 (35.4)	
CC (homozygous type)	84 (5.1)	281 (6.0)	
AMH (ng/ml)	3.34 (1.76, 5.75)	3.48 (1.76, 6.01)	0.319
Hemoglobin (g/l)	132 (126, 139)	133 (126, 139)	0.001
Fasting glucose (mmol/l)	4.8 (4.6, 5.1)	4.9 (4.5, 5.2)	0.302
Fasting insulin (μU/ml)	7.9 (5.7, 10.5)	8.1 (6.1, 10.7)	0.143
Triglyceride (mmol/l)	1.1 (0.8, 1.6)	1.2 (0.8, 1.8)	0.179
Total cholesterol (mmol/l)	5.1 (4.5, 5.9)	5.2 (4.6, 5.9)	0.733
LDL (mmol/l)	3.1 (2.7, 3.7)	3.2 (2.7, 3.7)	0.917
HDL (mmol/l)	1.5 (1.3, 1.8)	1.5 (1.3, 1,8)	0.352
Season of blood collection			<0.001
Spring	510 (31.1)	1,156 (24.6)	
Summer	407 (24.8)	1,464 (31.1)	
Autumn	372 (22.7)	1,319 (28.0)	
Winter	352 (21.5)	764 (16.2)	

Data are presented as median (Q1, Q3) or number (percentage). 25(OH)D, 25-hydroxyvitamin D; MTHFR, methylenetetrahydrofolate reductase; AMH, anti-mullerian hormone; LDL, low-density lipoprotein; HDL, high-density lipoprotein. Season of blood collection: Spring (March, April, May), Summer (June, July, Aug), Fall (Sept, Oct, Nov), Winter (Dec, Jan, Feb).

### MTHFR C677T and A1298C polymorphisms in infertile women

Table 2 summarizes the MTHFR C677T and A1298C genotypes and allele frequencies in infertile women. For MTHFR C677T, the prevalence of CC, CT and TT genotypes was 53.8, 37.5 and 8.7%, respectively, and the allele frequency was 0.73 for the C allele and 0.27 for the T allele. For MTHFR A1298C, the prevalence of AA, AC and CC genotypes was 59.2, 35.1 and 5.8%, respectively, and the allele frequency was 0.77 for the A allele and 0.23 for the C allele.

**Table 2:** The gene and allele frequencies of MTHFR C677T and A1298C.

Genotype and allele	Number	Frequencies (95% CI)	Allele frequency
MTHFR C677T			
CC (wild type)	3,410	53.8 (52.6–55.0)	
CT (heterozygous type)	2,381	37.5 (36.3–38.7)	
TT (homozygous type)	553	8.7 (8.0–9.4)	
Alleles			
C allele			0.73
T allele			0.27
MTHFR A1298C			
AA (wild type)	3,755	59.2 (58.0–60.4)	
AC (heterozygous type)	2,224	35.1 (33.9–36.3)	
CC (homozygous type)	365	5.8 (5.2–6.4)	
Alleles			
A allele			0.77
C allele			0.23

MTHFR, methylenetetrahydrofolate reductase; CI, confidence interval.

### Patient characteristics and blood biochemicals of MTHFR C677T and A1298C polymorphisms

We compared the clinical features and biochemical indicators of the MTHFR C677T genotypes and A1298C genotypes, respectively. Table 3 summarizes patient characteristics and biochemical parameters grouped by MTHFR C677T genotypes. Age, BMI, type of infertility, duration of infertility, AMH, hemoglobin, and a range of metabolic indicators did not differ among the three groups. Remarkably, patients with 677CT and TT genotypes had a significantly higher risk of vitamin D deficiency than those with CC genotype (27.8 and 29.7% vs. 23.9; *p* < 0.001). In addition, there were significant differences in the causes of infertility and Hcy levels between different C677T genotypes. As for MTHFR A1298C polymorphism, all of the above parameters, including vitamin D status and Hcy levels, did not differ significantly between A1298C genotypes (Supplementary Table S1).

**Table 3:** Patient characteristics and blood biochemicals grouped by MTHFR C677T genotypes.

Parameters	CC (*n* = 3,410)	CT (*n* = 2,381)	TT (*n* = 553)	*P-*value
Age ≥ 35 years	1,002 (29.4)	749 (31.5)	177 (32.0)	0.166
Body mass index	21.5 (19.8, 23.4)	21.6 (19.8, 23.6)	21.5 (19.7, 23.5)	0.414
Type of infertility				0.703
Primary	1724 (50.6)	1,181 (49.6)	272 (49.2)	
Secondary	1,686 (49.4)	1,200 (50.4)	281 (50.8)	
Duration of infertility	3 (1, 5)	2 (1, 5)	2.6 (1.0, 5.0)	0.412
Causes of infertility				0.002
Tubal	1,217 (35.7)^a^	805 (33.8)^a^	162 (29.3)^b^	
Male factor	479 (14.0)^a^	337 (14.2)^a^	117 (21.2)^b^	
Endometriosis	92 (2.7)	72 (3.0)	13 (2.4)	
PCOS	202 (5.9)	139 (5.8)	36 (6.5)	
Other	456 (13.4)	304 (12.8)	80 (14.5)	
More than one etiology	703 (20.6)	516 (21.7)	100 (18.1)	
Unexplained	261 (7.7)	208 (8.7)	45 (8.1)	
25(OH)D	62.4 (50.5, 75.7)^a^	61.4 (48.5, 74.9)^b^	61.0 (46.4, 74.3)^b^	0.003
Vitamin D deficiency*	816 (23.9)^a^	661 (27.8)^b^	164 (29.7)^b^	< 0.001
Homocysteine	7.1 (6.1, 8.1)^a^	7.3 (6.3, 8.3)^b^	7.5 (6.6, 8.8)^c^	< 0.001
AMH	3.44 (1.86, 5.91)	3.44 (1.64, 5.99)	3.39 (1.65, 5.88)	0.588
Hemoglobin	133 (126, 139)	133 (126, 139)	133 (125, 140)	0.879
Fasting glucose	4.8 (4.5, 5.1)	4.9 (4.5, 5.2)	4.9 (4.6, 5.2)	0.302
Fasting insulin	8.1 (6.1, 10.6)	7.9 (5.9, 10.7)	7.8 (5.7, 10.5)	0.232
Triglyceride	1.1 (0.8, 1.7)	1.1 (0.8, 1.7)	1.2 (0.9, 1.8)	0.138
Total cholesterol	5.2 (4.6, 5.9)	5.1 (4.5, 5.9)	5.2 (4.6, 5.9)	0.394
LDL	3.2 (2.7, 3.7)	3.1 (2.7, 3.6)	3.2 (2.7, 3.7)	0.415
HDL	1.5 (1.3, 1.8)	1.5 (1.3, 1.8)	1.5 (1.3, 1.7)	0.892

Data are presented as median (Q1, Q3) or number (percentage). MTHFR, methylenetetrahydrofolate reductase; 25(OH)D, 25-hydroxyvitamin D; AMH, anti-mullerian hormone; LDL, low-density lipoprotein; HDL, high-density lipoprotein. *Vitamin D deficiency: 25(OH)D < 50 nmol/l. ^a, b, c^ Different superscripts within the same line indicate statistical differences between subgroups.

**Figure 1:**
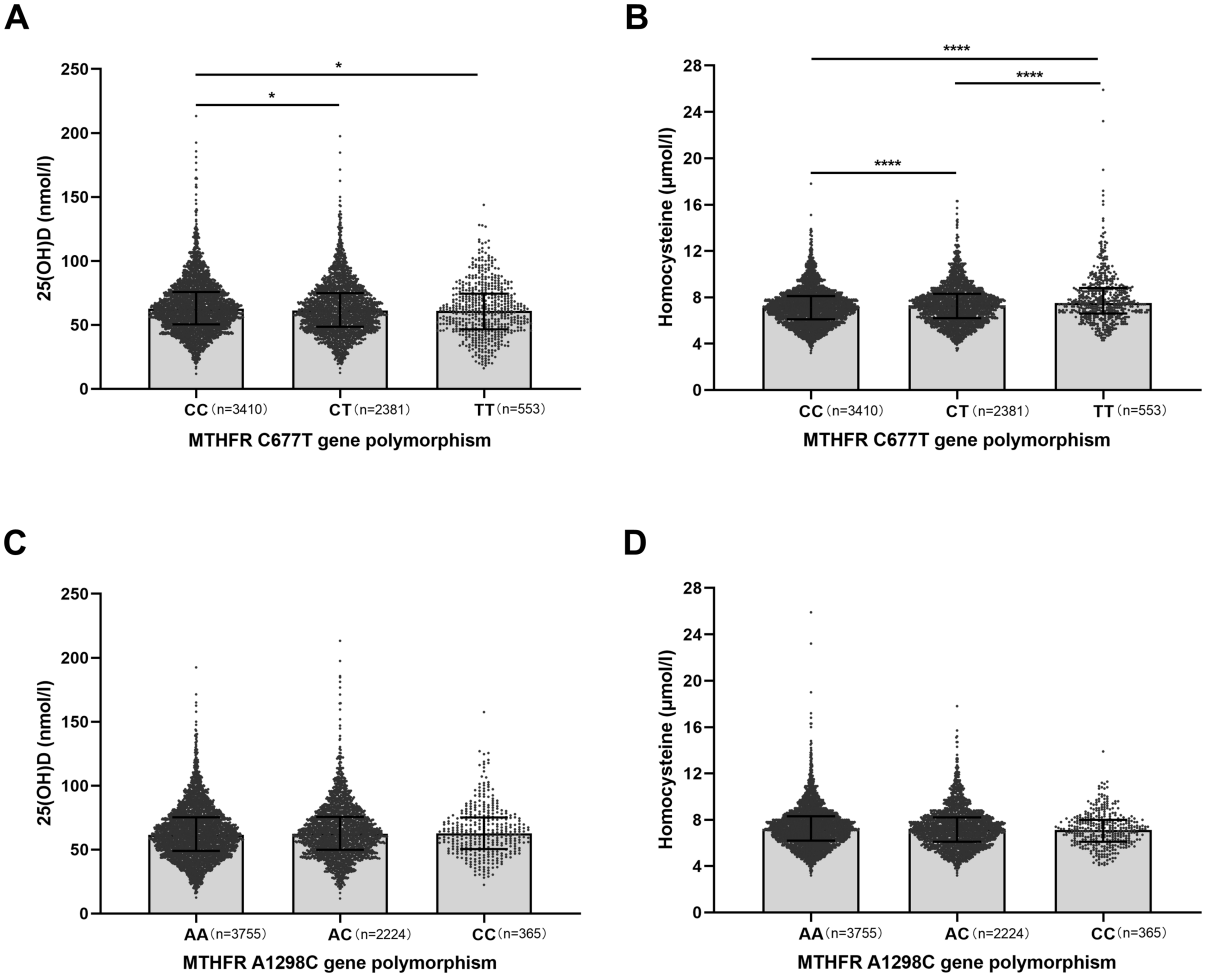
Effects of methylenetetrahydrofolate reductase (MTHFR) gene polymorphisms on serum 25(OH)D and homocysteine (Hcy) levels in infertile patients. **(A,B)** Effect of MTHFR C677T genotype (CC, CT, TT) on serum 25(OH)D (nmol/l) and Hcy (μmol/l) levels. CC, wild-type; CT, heterozygous; TT, homozygous. **(C,D)** Effect of MTHFR A1298C genotype (AA, AC, CC) on serum 25(OH)D and Hcy levels. AA, wild-type; AC, heterozygous; CC, homozygous. Data are shown as median with interquartile range. *p < 0.05, ****p < 0.0001.

Similarly, Figure 1 shows the effects of MTHFR C677T and A1298C polymorphisms on serum Hcy and 25(OH)D levels. In terms of C677T, Hcy levels in TT variant were significantly higher than those in CC and CT variants, and Hcy levels in CT variant were significantly higher than in CC variant. The 25(OH)D levels were significantly lower in TT and CT variants than in CC variant. In addition, A1298C polymorphisms did not affect Hcy and 25(OH)D levels (Figure 1).

### Association between MTHFR polymorphisms and vitamin D status

Multivariate logistic regression analyses were performed to assess the effect of MTHFR C677T and A1298C polymorphisms on vitamin D deficiency (Table 4). Based on prior knowledge and recommendation of DAG (Supplementary Figure S1), a set of covariates were selected and adjusted in the multivariate models. When Hcy was the mediating variable, two models were built based on DAG. In model 1, after controlling for age, BMI, AMH, type of infertility and causes of infertility, MTHFR 677CT (adjusted OR, 1.225; 95% CI, 1.087–1.380) and TT (adjusted OR, 1.355; 95% CI, 1.110–1.654) were positively associated with the risk of vitamin D deficiency compared with CC. In model 2, MTHFR 677CT (adjusted OR, 1.229; 95% CI, 1.089–1.386) and TT (adjusted OR, 1.355; 95% CI, 1.109–1.657) were also significantly associated with the risk of vitamin D deficiency compared with CC. With regard to MTHFR A1298C, there were no significant effects of different polymorphisms on vitamin D deficiency in the two logistic regression models (Table 4).

**Table 4:** Multivariable logistic regression analysis for the effect of MTHFR polymorphisms on vitamin D deficiency.

Parameters	Model 1			Model 2		
	Adjusted OR	95% CI	*P* value	Adjusted OR	95% CI	*P* value
MTHFR C677T			< 0.001			< 0.001
CC (wild type)	Reference			Reference		
CT (heterozygous type)	1.225	1.087–1.380	0.001	1.229	1.089–1.386	0.001
TT (homozygous type)	1.355	1.110–1.654	0.003	1.355	1.109–1.657	0.003
MTHFR A1298C			0.198			0.17
AA (wild type)	Reference			Reference		
AC (heterozygous type)	0.925	0.820–1.043	0.203	0.919	0.814–1.037	0.171
CC (homozygous type)	0.826	0.640–1.066	0.142	0.821	0.635–1.061	0.131

Vitamin D deficiency: 25(OH)D < 50 nmol/l. OR, odds ratio; CI, confidence interval; MTHFR, methylenetetrahydrofolate reductase. Model 1 was adjusted for age, body mass index, AMH, type of infertility, causes of infertility, and MTHFR genotypes. Model 2 was adjusted for age, body mass index, AMH, type of infertility, causes of infertility, MTHFR genotypes, hemoglobin, and season of blood collection.

Multiple linear regression analyses were used to assess the effect of MTHFR C677T and A1298C polymorphisms on serum 25(OH)D levels (Table 5). In model 1, MTHFR 677CT (B, −1.371; 95%CI, −2.448, −0.293) and TT (B, −2.799; 95%CI, −4.652, −0.946) were negatively correlated with serum 25(OH)D levels compared to CC. In model 2, MTHFR C677T genotypes had a similar effect on 25(OH)D levels. As well, the MTHFR A1298C genotypes had no significant effect on 25(OH)D levels in either model.

**Table 5:** Multiple linear regression analysis for the effect of MTHFR polymorphisms on serum 25(OH)D levels.

Parameters	**Model 1**				**Model 2**			
	Unstandardized coefficients		Standardized coefficients	*p-*value	Unstandardized coefficients		Standardized coefficients	*p-*value
	B	95% CI	*β*		B	95%CI	*β*	
MTHFR C677T								
CC (wild type)	Reference				Reference			
CT (heterozygous type)	−1.371	−2.448, −0.293	−0.032	0.013	−1.387	−2.455, −0.319	−0.033	0.011
TT (homozygous type)	−2.799	−4.652, −0.946	−0.038	0.003	−2.75	−4.585, −0.914	−0.038	0.003
MTHFR A1298C								
AA (wild type)	Reference				Reference			
AC (heterozygous type)	0.991	−0.090, 2.071	0.023	0.072	1.036	−0.035, 2.107	0.024	0.058
CC (homozygous type)	1.173	−1.041, 3.387	0.013	0.299	1.252	−0.942, 3.445	0.014	0.263

CI, confidence interval; 25(OH)D, 25-hydroxyvitamin D; MTHFR, methylenetetrahydrofolate reductase. Model 1 was adjusted for age, body mass index, AMH, type of infertility, causes of infertility, and MTHFR genotypes. Model 2 was adjusted for age, body mass index, AMH, type of infertility, causes of infertility, MTHFR genotypes, hemoglobin, and season of blood collection.

Due to the significant difference in age between the two vitamin D strata, we divided all patients into two subgroups: age < 35 years and ≥35 years, and separately performed multivariable logistic regression analyses (Supplementary Table S2). In multivariable analysis of patients aged < 35 years, MTHFR 677CT (adjusted OR, 1.227; 95% CI, 1.064–1.414) and TT (adjusted OR, 1.421; 95% CI, 1.121–1.802) were positively associated with the risk of vitamin D deficiency compared with CC. However, the effect of C677T on vitamin D deficiency was not significant in multivariable analysis of patients aged ≥ 35 years. For A1298C polymorphism, the 1,298 AC (heterozygous) genotype was negatively associated with vitamin D deficiency in the younger subgroup, whereas in the old subgroup, the A1298C polymorphism was not associated with vitamin D deficiency (Supplementary Table S2).

### Association between MTHFR polymorphisms, vitamin D deficiency and Hcy

**Figure 2:**
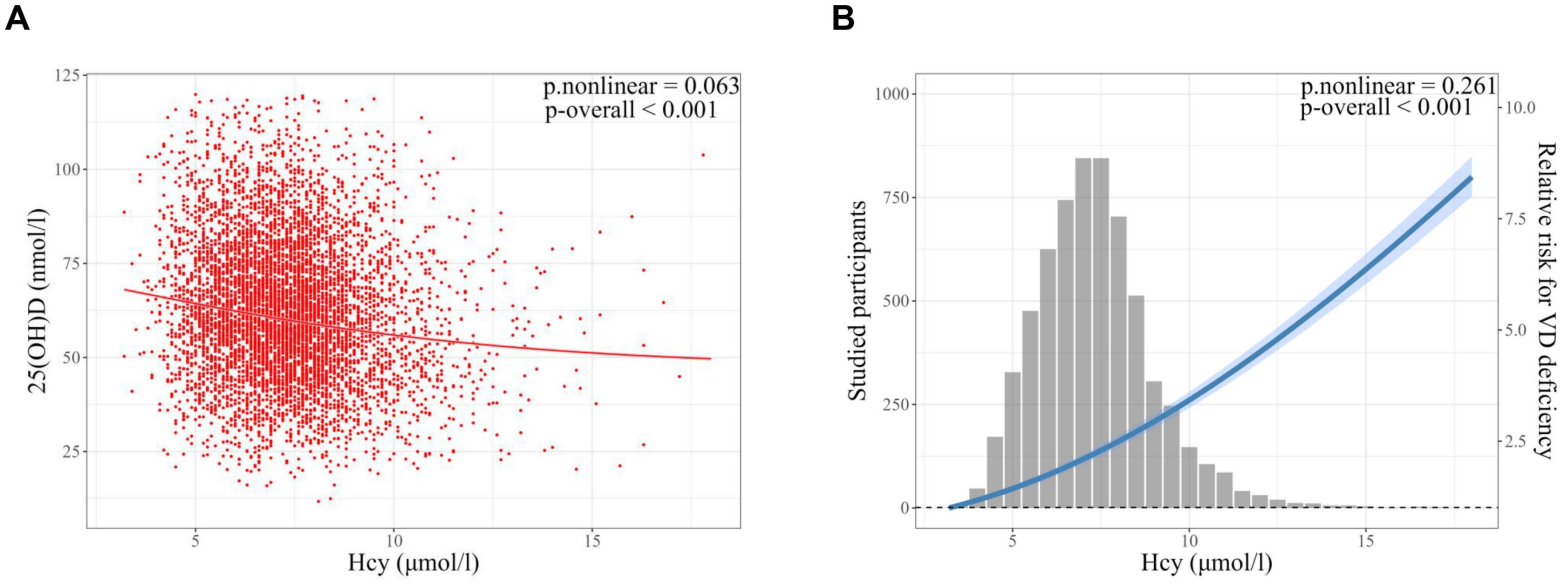
Smooth curve fitting models of the correlation between serum homocysteine (Hcy) and vitamin D (VD) status in infertile patients. **(A)** Smooth curve fitting of the correlation between serum Hcy (μmol/l) and 25(OH)D (nmol/l) levels. **(B)** Smooth curve fitting of the correlation between serum Hcy and vitamin D deficiency (25(OH)D < 50 nmoL/L). Models were adjusted for age, body mass index, AMH, type of infertility, causes of infertility, MTHFR C677T genotype, hemoglobin, and season of blood collection. Sample size: *n* = 6,344. Hcy, homocysteine; VD, vitamin D. *p*-overall, *p*-value for model test; *p*-nonlinear, *p*-value for nonlinear test.

The smooth curve fitting showed a linear correlation between serum Hcy and 25(OH)D levels (*p*-overall < 0.001; *p*-nonlinear = 0.063) (Figure 2A). Moreover, serum Hcy was linearly associated with the risk of vitamin D deficiency (*p*-overall < 0.001; *p*-nonlinear = 0.261) (Figure 2B). The correlation coefficients between serum Hcy and 25(OH)D levels were calculated by spearman correlation analysis for the total population and for different MTHFR C677T genotypes (Supplementary Figure S2). A negative correlation between Hcy and 25(OH)D levels was observed in the total population (*R* = −0.137, *p* < 0.001). A greater negative correlation between Hcy and 25(OH)D levels was observed in the TT genotype population compared to total, CC and CT genotype populations (Supplementary Figure S2).

**Figure 3:**
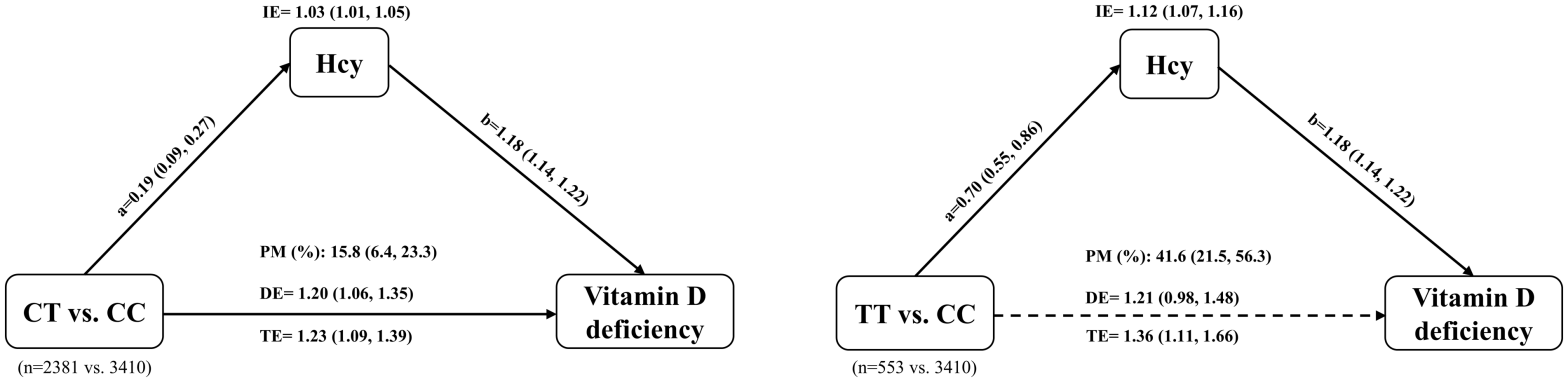
Mediation effect of homocysteine (Hcy) in the association between MTHFR C677T genotype and vitamin D deficiency. The total effect (TE) of the C677T genotype on vitamin D deficiency is partitioned into a direct effect (DE) and an indirect effect (IE) mediated through Hcy. Path ‘a’ represents the estimated change in Hcy levels for the CT or TT genotype compared with the CC genotype. Path ‘b’ represents the estimated change in the risk of vitamin D deficiency for each unit increase in Hcy. The dotted arrow indicates that the direct effect was not statistically significant. The Models were adjusted for age, BMI, AMH, type of infertility, causes of infertility, hemoglobin, and season of blood collection. IE, indirect effect; DE, direct effect; TE, total effect; PM, proportion mediated; CC, wild type; CT, heterozygous type; TT, homozygous type.

Hypothesizing that MTHFR C677T may affect vitamin D status through the homocysteine metabolic pathway, we used mediation analysis to partition the total effect of C677T on vitamin D status into a direct effect and an indirect effect mediated by Hcy. Figure 3 shows the mediating effect of Hcy on the association between C677T genotypes and vitamin D deficiency. The total effect of CT vs. CC on vitamin D deficiency was significant (OR, 1.23; 95% CI, 1.09, 1.39). After controlling for Hcy, the direct effect of CT vs. CC on vitamin D deficiency was dominant (OR, 1.20; 95% CI, 1.06, 1.35). The indirect effect of CT vs. CC on vitamin D deficiency mediated by Hcy was also significant (OR, 1.03; 95% CI, 1.01, 1.05), with mediation proportion of 15.8% (95% CI, 6.4, 23.3%). Furthermore, the total effect of TT vs. CC on vitamin D deficiency was more significant (OR, 1.36; 95% CI, 1.11, 1.66). Of note, the direct effect of TT versus CC on vitamin D deficiency was not statistically significant (OR, 1.21; 95% CI, 0.98, 1.48), although the confidence interval indicates a trend toward a positive association. The indirect effect mediated by Hcy was remarkable (OR, 1.12; 95% CI, 1.07, 1.16), with mediation proportion of 41.6% (95% CI, 21.5, 56.3%) (Figure 3). In addition, when analyzing 25(OH)D levels as the outcome, we observed consistent associations (Supplementary Figure S3). Hcy mediated 20.4% of the effect of CT vs. CC on 25(OH)D levels and 39.9% of the effect of TT vs. CC on 25(OH)D levels.

## Discussion

MTHFR regulates folate metabolism and homocysteine methylation (1), which is strongly associated with female reproductive health. Both folate deficiency (50–52) and vitamin D deficiency (31–34) are closely related to adverse pregnancy outcomes and female reproductive disorders. In this study, we investigated whether two genetic MTHFR polymorphisms (C677T and A1298C) are associated with an increased risk of vitamin D deficiency, and further examined the mediation effects of Hcy.

We found that infertile patients with heterozygous 677CT and homozygous TT genotypes were significantly more likely to be vitamin D deficient than wild-type patients. The risk of vitamin D deficiency and serum Hcy levels were significantly higher in females with heterozygote (677CT) and homozygote (677TT) compared with wild type. There were no significant differences found for A1298C polymorphism. Because correlations have been found between C677T and vitamin D, between C677T and Hcy, and between Hcy and vitamin D, we employed mediation analysis to decompose the total effect of C677T on vitamin D status into a direct effect and an indirect effect mediated by Hcy. The total effect of CT vs. CC on vitamin D deficiency consisted of direct and indirect effects, with 15.8% mediated by Hcy. Intriguingly, the direct effect of TT vs. CC on vitamin D deficiency was not significant, whereas the indirect effect mediated by Hcy was evident, with a mediation proportion of 41.6%. This suggests that for TT genotype, the mediating role of Hcy dominates the total effect of TT vs. CC on vitamin D deficiency.

There is a lack of evidence regarding the relationship between MTHFR polymorphisms and vitamin D status. Our results showed that C677T polymorphism was significantly associated with vitamin D deficiency in infertile women. Conversely, studies have shown no significant difference in serum 25(OH)D levels between MTHFR C677T genotypes in perimenopausal women (53) and healthy young men (54). Consistent with our findings, Ota et al. (45) found that serum 25(OH)D levels were lower in TT genotype than in wild-type in patients with RPL. However, their study had a small sample size (*n* = 837), included only women with RPL, and may not be generalizable to other populations. They did not analyze the relationship between MTHFR 1298 polymorphism and vitamin D status. Besides, they did not address the specific relationship between MTHFR polymorphisms, Hcy, and vitamin D and the extent of their correlation.

Many studies have found that MTHFR C677T gene polymorphism is associated with Hcy levels in various populations (4, 45, 55), whereas the correlation between A1298C polymorphism and Hcy levels is not as significant as that of C677T (56, 57), and our results are in line with previous studies. Previous studies have found a negative correlation between 25(OH)D and Hcy levels in the general population and in women PRL (44, 45), and both vitamin D deficiency and hyperhomocysteinemia are risk factors for atherosclerotic disease (23, 30) and adverse pregnancy outcomes (31, 58). Consistent with previous studies, our results showed that serum 25(OH)D levels were inversely correlated with Hcy levels, with a higher degree of negative correlation in the 677TT genotype population than in the CC genotype and total populations. These studies showed that vitamin D deficiency and elevated Hcy coexist in different populations and diseases.

To our knowledge, this is the first study with a large sample size in infertile women to demonstrate a significant correlation between MTHFR C677T polymorphisms and vitamin D status, while also quantifying the mediating role of Hcy. MTHFR polymorphisms, hyperhomocysteinemia, and vitamin D deficiency have all been reported as risk factors for infertility and adverse pregnancy outcomes (25–28, 31–34). Our findings reveal that the C677T polymorphism is significantly associated with an increased risk of vitamin D deficiency in infertile women, with part of this effect mediated by elevated Hcy levels. These results have potential clinical implications. Given the critical roles of folate and vitamin D in female reproduction, screening for MTHFR C677T variants may be valuable in infertility evaluations. Early identification of individuals with impaired folate metabolism may guide personalized interventions, such as supplementation with active folate and vitamin D. In addition, Hcy may serve as a modifiable intermediary target to improve reproductive outcomes. Our mediation analysis suggests both direct and indirect effects of C677T on vitamin D status, with the indirect effect through Hcy particularly pronounced in TT carriers. These findings underscore the complex interplay between genetic, metabolic, and nutritional factors in reproductive health and highlight the need for further prospective studies to validate targeted intervention strategies.

Our study highlights the possible interrelationship between folate metabolism and vitamin D. On the one hand, folate metabolism pathway may influence vitamin D status. Previous studies showed that vitamin D biosynthesis in the skin was related to folate metabolism (59) and that folic acid supplementation significantly increased vitamin D levels in eggs of laying hens, but had no effect on vitamin A and vitamin E levels (60). Although these findings may not be directly applicable to humans, they provide preliminary mechanistic insight that warrants further investigation in human studies. On the other hand, vitamin D may affect folate metabolism pathway and downstream functions. Vitamin D has been reported to reduce Hcy levels in cell culture *in vitro* (61) and in overweight reproductive women (62), suggesting that vitamin D may regulate gene expression of enzymes involved in homocysteine metabolism. Nonetheless, the exact mechanism of the association between folate metabolism pathway and vitamin D is unknown and needs further study.

Some studies have shown that increasing the dose of folic acid may be ineffective or even harmful to the mother and offspring (27, 28), and vitamin D supplementation was not beneficial in several clinical trails (63, 64), but the underlying reasons for these negative results are not known. Previous research has shown that vitamin D regulatory pathways have much in common with folate metabolism-related pathways, including inflammation, immunomodulation, and various metabolic processes (38–40). From this, we hypothesized that folic acid supplementation alone may not fully ameliorate disorders associated with abnormal folate metabolism, including cardiovascular disease and female reproductive disorders, and that combined supplementation with folic acid and vitamin D may be more beneficial in population with C677T polymorphism, as they may synergistically affect folate metabolism pathway and downstream function. So far, there have been no studies on combined folic acid and vitamin D supplements in humans, and future research is warranted. Routine monitoring of vitamin D and homocysteine enables personalized supplementation strategies by identifying subclinical deficiencies, guiding genotype-specific interventions, and preventing metabolic cascades detrimental to fertility. This dual biomarker approach based on mechanistic insights, transforms pre-conception care from reactive nutrient replacement to proactive, precise metabolic optimization, ultimately improving reproductive success and perinatal health.

There are some limitations of this study. Due to the retrospective nature of the study, some patient characteristics—such as smoking habits, physical activity, and the dose and duration of folic acid supplementation—could not be obtained. Although the infertile women in this study had initiated folic acid supplementation in preparation for pregnancy, variations in the dose and timing may have introduced confounding that could not be accounted for in this analysis. This limitation may also affect the accuracy of the estimated mediation effects. Future prospective studies with standardized collection of supplement usage data are warranted to clarify these relationships more precisely. In addition, the relationship between MTHFR polymorphisms and serum 25(OH)D levels was investigated in infertile women, not in normal fertile or menopausal women, so our results may not be generalizable to unselected populations. Moreover, there was a significant difference in age between the two vitamin D strata in this study, which is consistent with our previous report (34). To further exclude the interference of age, all patients were divided into two subgroups based on age threshold. In the younger subgroup, MTHFR C677T polymorphism was positively associated with vitamin D deficiency, whereas in the older subgroup, C677T polymorphism was not associated with vitamin D deficiency. In agreement with our findings, a previous study found that serum 25(OH)D3 levels did not differ between MTHFR C677T genotypes in perimenopausal women (45–58 years) (53). Besides, the 1,298 AC (heterozygous) genotype was negatively correlated with vitamin D deficiency in the younger subgroup, while the A1298C polymorphism was not correlated with vitamin D deficiency in the old subgroup. Therefore, the effect of MTHFR polymorphisms on vitamin D deficiency needs to be interpreted separately according to different age groups. However, we have no clear explanation for this phenomenon and speculate that the effect of MTHFR polymorphisms on vitamin D status is likely to be greater in younger population, whereas in older population, vitamin D status may be related to more factors other than MTHFR polymorphisms. We hypothesize that folic acid and/or vitamin D supplementation may be more effective in correcting the potential adverse effects of MTHFR polymorphisms in younger population compared to older population. Caution should be exercised in interpreting our findings and, given these limitations, external validation of the study is needed in the future.

## Conclusion

In summary, both direct and Hcy-mediated pathways may contribute to the link between MTHFR C677T polymorphisms and vitamin D deficiency in infertile women. We hypothesize that elevated Hcy and vitamin D deficiency in infertile patients with MTHFR C677T polymorphism may together contribute to the development of diseases associated with abnormal folate metabolism. Further research into the mechanisms underlying the interrelationship between folate metabolism and vitamin D is needed, and clinical trials of combined folate and vitamin D supplementation are urgently required.

## Data Availability

The raw data supporting the conclusions of this article will be made available by the authors, without undue reservation.
