# Supplementary material for: Association between MTHFR polymorphisms and vitamin D status in infertile women: a mediation analysis
Source: Front Nutr. 2025 Sep 11;12:1644302. doi: 10.3389/fnut.2025.1644302 (PMC12460134; doi:10.3389/fnut.2025.1644302)
Supplement: Supplementary file 1 [file Supplementary_file_1.docx]

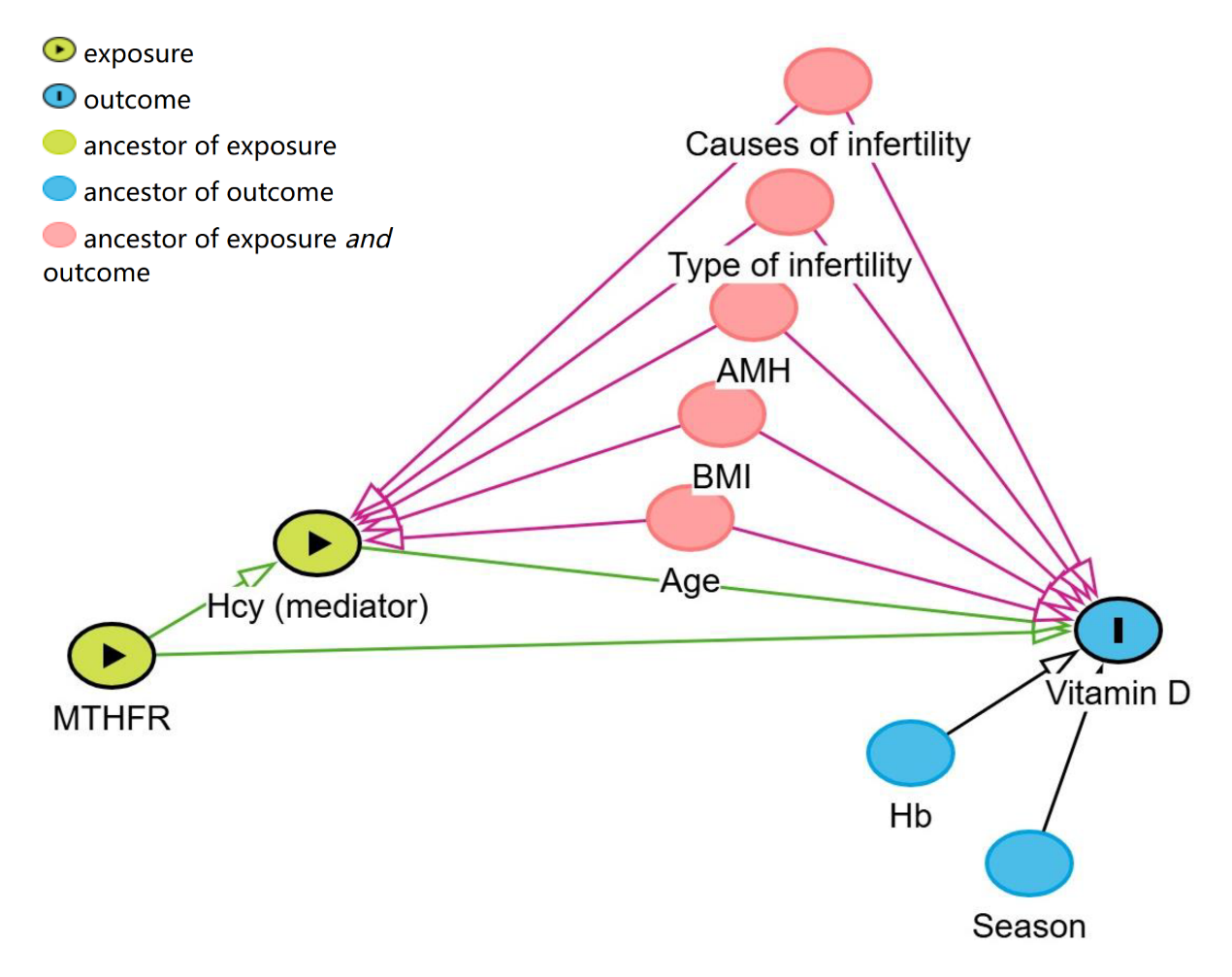
**Figure S1.** Directed acyclic graph (DAG) representation of possible confounders drawn by DAGitty software. Two models were developed based on DAG, with Hcy as the mediating variable. In model 1, minimal sufficient adjusted variables included age, BMI, AMH, type of infertility and causes of infertility to estimate the total effect of MTHFR polymorphisms on vitamin D status. In model 2, age, BMI, AMH, type of infertility, causes of infertility, hemoglobin and season of blood collection were adjusted confounders to estimate the total effect of MTHFR polymorphisms on vitamin D status. MTHFR, methylenetetrahydrofolate reductase; Hcy, homocysteine; BMI, body mass index; AMH, anti-mullerian hormone; Hb, hemoglobin.


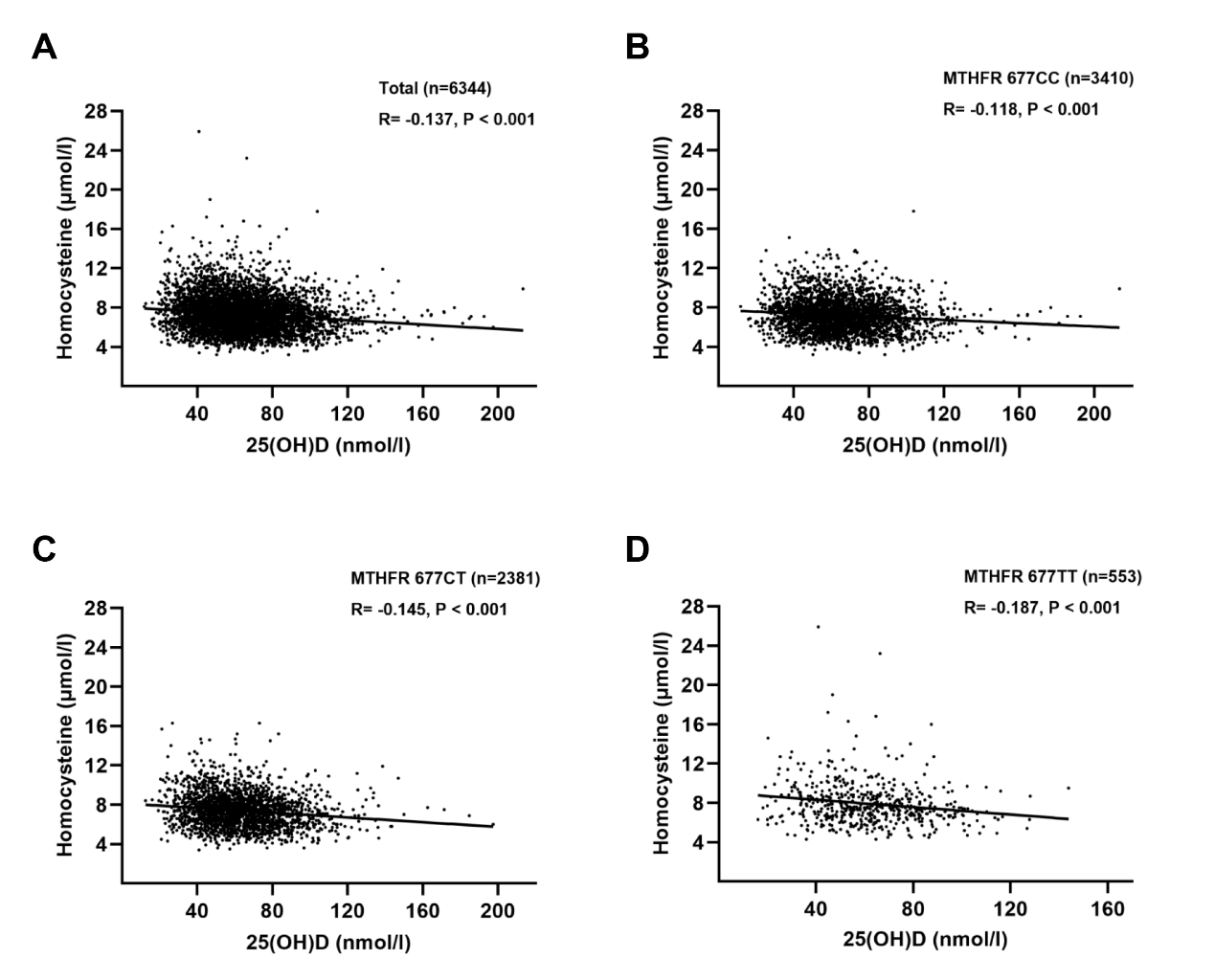


**Figure S2.** Effects of MTHFR C677T polymorphism on the correlation between serum homocysteine levels and 25(OH)D levels in infertile patients. Spearman correlation analyses between serum homocysteine levels and 25(OH)D levels in total polulation (A), CC (wild type) (B), CT (heterozygous type) (C), and TT (homozygous type) (D).


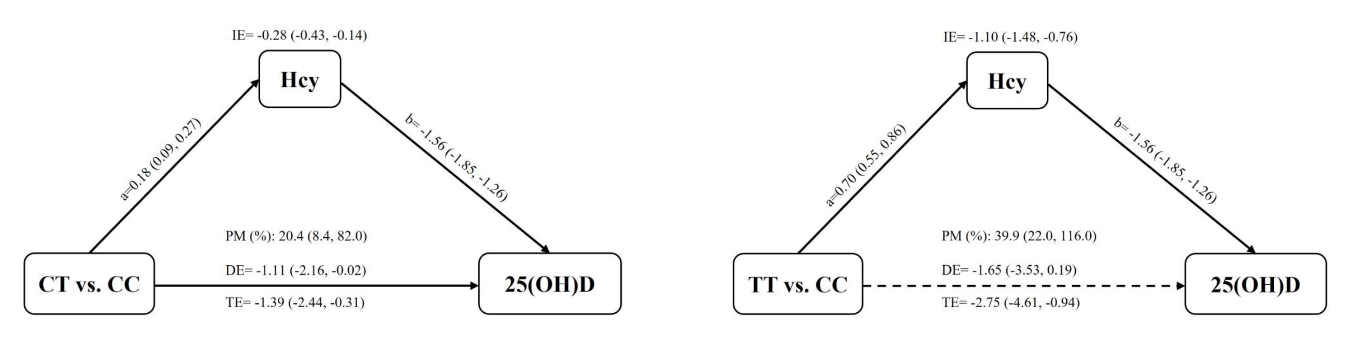


**Figure S3.** Mediation effect of homocysteine (Hcy) in the association between MTHFR C677T genotype and serum 25(OH)D levels. The total effect of C677T genotype on serum 25(OH)D levels consisted of direct and indirect effects. Models were adjusted for age, body mass index, AMH, type of infertility, causes of infertility, hemoglobin, and season of blood collection. a was an estimate of each unit increase in Hcy for CT or TT compared with CC, b was an estimate of serum 25(OH)D levels for each unit increase in Hcy. CC, wild type; CT, heterozygous type; TT, homozygous type; IE, indirect effect; DE, direct effect; TE, total effect; PM, proportion mediated. The dotted line stands for the direct effect was not significant.
