# Supplementary material for: Association between MTHFR polymorphisms and vitamin D status in infertile women: a mediation analysis
Source: Front Nutr. 2025 Sep 11;12:1644302. doi: 10.3389/fnut.2025.1644302 (PMC12460134; doi:10.3389/fnut.2025.1644302)
Supplement: Supplementary file 2 [file Table_1.docx]

**Table S1** Patient characteristics and blood biochemicals grouped by MTHFR A1298C genetypes.

|  | **AA (n=3755)** | **AC (n=2224)** | **CC (n=365)** | ***P* value** |
| --- | --- | --- | --- | --- |
| Age ≥ 35 years | 1128 (30.0) | 686 (30.8) | 114 (31.2) | 0.757 |
| Body mass index  Type of infertility  Primary  Secondary  Duration of infertility  Causes of infertility  Tubal  Male factor  Endometriosis  PCOS  Other  More than one etiology  Unexplained  25(OH)D  Vitamin D deficiency*  Homocysteine | 21.5 (19.8, 23.4)  1879 (50.0)  1876 (50.0)  2.5 (1.0, 4.4)  1290 (34.4)  570 (15.2)  105 (2.8)  204 (5.4)  495 (13.2)  768 (20.5)  323 (8.6)  61.6 (49.0, 75.4)  1000 (26.6)  7.3 (6.3, 8.3) | 21.5 (19.8, 23.5)  1119 (50.3)  1105 (49.7)  3.0 (1.0, 5.0)  763 (34.3)  318 (14.3)  62 (2.8)  155 (7.0)  294 (13.2)  470 (21.1)  162 (7.3)  62.1 (50.2, 75.5)  557 (25.0)  7.2 (6.2, 8.2) | 21.7 (19.8, 23.8)  179 (49.0)  186 (51.0)  3.0 (1.5, 5.0)  131 (35.9)  45 (12.3)  10 (2.7)  18 (4.9)  51 (14.0)  81 (22.2)  29 (7.9)  62.3 (50.8, 74.9)  84 (23.0)  7.1 (6.1, 8.0) | 0.176  0.901  0.115  0.404  0.168  0.176  0.059 |
| AMH  Hemoglobin  Fasting glucose  Fasting insulin  Triglyceride  Total cholesterol  LDL  HDL | 3.44 (1.80, 5.90)  133 (126, 139)  4.9 (4.5, 5.2)  8.1 (6.0, 10.7)  1.1 (0.8, 1.7)  5.1 (4.5, 5.8)  3.2 (2.7, 3.6)  1.5 (1.3, 1.8) | 3.40 (1.72, 6.01)  133 (126, 139)  4.9 (4.5, 5.1)  8.0 (6.0, 10.5)  1.2 (0.8, 1.8)  5.2 (4.6, 6.0)  3.2 (2.7, 3.7)  1.5 (1.3, 1.8) | 3.61 (1.85, 5.95)  134 (125, 140)  4.8 (4.5, 5.1)  7.7 (5.9, 10.6)  1.2 (0.8, 1.8)  5.2 (4.6, 6.0)  3.2 (2.7, 3.8)  1.5 (1.3, 1.8) | 0.731  0.535  0.155  0.720  0.645  0.234  0.128  0.955 |

Data are presented as median (Q1, Q3) or number (percentage). 25(OH)D, 25-hydroxyvitamin D; MTHFR, methylenetetrahydrofolate reductase; AMH, anti-mullerian hormone; LDL, low-density lipoprotein; HDL, high-density lipoprotein.

*Vitamin D deficiency: 25(OH)D < 50 nmol/l.

**Table S2** Multivariable logistic regression analysis of the effect of MTHFR polymorphisms on vitamin D deficiency for subgroups of patients aged < 35 and ≥ 35 years.

|  | **Patients age < 35 years** | | **Patients age** ≥ **35 years** | | |
| --- | --- | --- | --- | --- | --- |
| **Parameters** | **Adjusted OR (95%CI)** | ***P* value** | | **Adjusted OR (95%CI)** | ***P* value** |
| MTHFR C677T  CC (wild type)  CT (heterozygous type)  TT (homozygous type)  MTHFR A1298C  AA (wild type)  AC (heterozygous type)  CC (homozygous type) | Reference  1.227 (1.064-1.414)  1.421 (1.121-1.802)  Reference  0.862 (0.746-0.995)  0.854 (0.633-1.153) | 0.002  0.005  0.004  0.101  0.043  0.303 | | Reference  1.236 (0.985-1.551)  1.187 (0.808-1.744)  Reference  1.078 (0.859-1.354)  0.712 (0.431-1.175) | 0.174  0.068  0.381  0.276  0.515  0.184 |

Vitamin D deficiency: 25(OH)D < 50 nmol/l.

OR, odds ratio; CI, confidence interval; MTHFR, methylenetetrahydrofolate reductase.

Models adjusted for BMI, AMH, type of infertility, causes of infertility, MTHFR genotypes, hemoglobin, and season of blood collection.
